# Thoracic Trauma: Current Approach in Emergency Medicine

**DOI:** 10.3390/clinpract14050148

**Published:** 2024-09-10

**Authors:** Giorgia Caputo, Stefano Meda, Andrea Piccioni, Angela Saviano, Veronica Ojetti, Gabriele Savioli, Gaia Bavestrello Piccini, Chiara Ferrari, Antonio Voza, Lavinia Pellegrini, Miriam Ottaviani, Federica Spadazzi, Gianpietro Volonnino, Raffaele La Russa

**Affiliations:** 1 Division of Anesthesia and Critical Care, Santi Antonio e Biagio e Cesare Arrigo Hospital, 15121 Alessandria, Italy; giorgia.caputo@ymail.com; 2 Division of Thoracic Surgery, Santi Antonio e Biagio e Cesare Arrigo Hospital, 15121 Alessandria, Italy; smeda@ospedale.al.it; 3 Department of Emergency Medicine, Fondazione Policlinico Universitario A. Gemelli IRCCS, 00168 Rome, Italy; andrea.piccioni@policlinicogemelli.it (A.P.); angela.saviano@policlinicogemelli.it (A.S.); 4 Internal Medicine Department, San Carlo di Nancy Hospital, 00165 Rome, Italy; 5 Emergency Medicine and Surgery, IRCCS Fondazione Policlinico San Matteo, 27100 Pavia, Italy; gabrielesavioli@gmail.com; 6 Emergency Medicine Residency Program, Université Libre de Bruxelles, 1050 Brussels, Belgium; gaia.bavestrellopic01@universitadipavia.it; 7 Division of Anesthesia, Intensive Care, Pain Medicine, Policlinico Hospital, 70124 Bari, Italy; ferrari_chiara@live.it; 8 Emergency Medicine, IRCCS Humanitas Research Hospital, Rozzano, 20089 Milan, Italy; antonio.voza@humanitas.it; 9 Department of Biomedical Sciences, Humanitas University, Via Rita Levi Montalcini 4, Pieve Emanuele, 20072 Milan, Italy; 10 Department of Anatomical, Histological, Forensic and Orthopaedic Sciences, Sapienza University of Rome, Viale Regina Elena 336, 00161 Rome, Italy; lavinia.pellegrini@uniroma1.it (L.P.); miriam.ottaviani@uniroma1.it (M.O.); federica.spadazzi@uniroma1.it (F.S.); gianpietro.volonnino@uniroma1.it (G.V.); 11 Department of Clinical Medicine, Public Health, Life Sciences, Environmental Sciences, University of L’Aquila, 67100 L’Aquila, Italy

**Keywords:** thoracic trauma, blunt injury, penetrating injury, risk management, critical care, surgery, emergency department thoracotomy, prehospital thoracotomy, thoracic blunt and penetrating trauma

## Abstract

Chest trauma is the leading cause of death in people under 40. It is estimated to cause around 140,000 deaths each year. The key aims are to reduce mortality and the impact of associated complications to expedite recovery and to restore patient’s conditions. The recognition of lesions through appropriate imaging and early treatment already in the emergency department are fundamental. The majority can be managed in a non-surgical way, but especially after traumatic cardiac arrest, a surgical approach is required. One of the most important surgical procedures is the Emergency Department Thoracotomy (EDT). The aim of this review is to provide a comprehensive synthesis about the management of thoracic trauma, the surgical procedures, accepted indications, and technical details adopted during the most important surgical procedures for different thoracic trauma injuries. Literature from 1990 to 2023 was retrieved from multiple databases and reviewed. It is also important to emphasize the medico-legal implications of this type of trauma, both from the point of view of collaboration with the judicial authority and in the prevention of any litigation.

## 1. Introduction

Chest trauma is the leading cause of death under 40. It is estimated to cause around 140,000 deaths each year [1]. In total, 75% of cases are represented by penetrating and blunt injuries [2,3]. Patients are usually primarily treated by chest tube placement [4], while 10–15% will receive definitive surgical indication [5]. Surgical timing, crucial for this type of trauma, is strongly influenced by the patient’s clinical condition. Immediate treatment is thoracotomy in the emergency room; in an interval of 1–4 h, the patient should undergo surgery, postponed to a maximum of 24 h after hospitalization [6,7,8]. The site of the injury, together with the type of trauma, dictate the choice of surgical technique and its access route.

The diagnosis and treatment of thoracic trauma have been extensively described over the years. The terms “stove in chest” and “flail chest” were first used in publications since 1945. Patients with severe chest wall injuries were treated with long-term positive mechanical ventilation or “internal pneumatical stabilization” during the 1960s and 1970s.

This idea was contested in 1976 by Trinkel, Shackford and colleagues [8]. Consequently, mechanical ventilation was partially abandoned.

Over the past century, many surgical methods to fix chest injuries were described; despite this, nonsurgical methods have mainly been used.

Over the last twenty years, there has been an important increase in interest in the surgical management of thoracic trauma. This is probably due to the findings of multiple authors who have demonstrated better results in the most severely wounded patients as compared to traditional care.

The purpose of the review is the examination of the algorithm commonly used for the management of thoracic trauma. The various scenarios will be evaluated considering each characteristic and peculiarities that condition the decision-making process. It is important to underline that chest trauma can be a consequence of events of legal and medico-legal interest, such as traffic accidents, murders, and therefore an adequate management of individual cases by health professionals will also be fundamental, with particular attention to the description and documentation of injuries.

### 1.1. Initial Management, Primary and Secondary ATLS Survey

During the first management, also known as the “primary survey” of the injured trauma patient, the identification of significant intrathoracic and chest wall traumas is crucial. IV access should be immediately performed in severe injuries [9]

Following attention to airway, breathing and circulation, a history should be taken if possible. A physical examination with a focus on quickly recognizing life-threatening illnesses should then be conducted [10].

Examiners should pay attention to chest rise, searching for any noticeable asymmetry that may suggest a flail segment. Examiners may also palpate the chest wall, checking for symmetry, crepitance or even movable segments. Auscultation is also necessary but could be challenging in an emergency room. It is critical to recognize major injuries affecting the airway during the primary survey, but airway management is one of the most important things to evaluate. It is recommended to evaluate a drug-assisted rapid sequence induction of anesthesia and intubation [11].

It is important to regularly monitor vital signs; it is important to consider that patients with severe thoracic trauma are usually polytrauma patients. In addition, arterial blood gas, complete blood count, platelets, electrolytes, cardiac markers, serum lactate levels, blood typing and a coagulation test should be requested. Hypovolemia and vascular collapse in the trauma patient are associated with mortality, so is important to be avoided and appropriate volume resuscitation and acidosis treatment are very important in these patients.

If the patient is hemodynamically stable and has a severe chest wall injury and a possible pulmonary contusion, the use of crystalloid fluid resuscitation should be used carefully. It is wise to avoid administering intravenous fluid unnecessarily even though there is not strong evidence that the volume of crystalloid correlates with poor outcomes [12].

### 1.2. EFAST Ultrasound and Radiographic Diagnosis before Surgical Procedures

The Extended Focused Assessment Using Sonography for Trauma (EFAST) examination, performed at bedside or in the pre-hospital setting, has become a standard of care diagnostic procedure in trauma patients [13,14].

EFAST can identify fluid within the peritoneal, pericardial and pleural cavities, which is usually blood, but also cardiac tamponade, PEA, by detecting cardiac motion. The detection of cardiac motions is very important in patients with a traumatic cardiac arrest with no vital signs, because it can provide crucial information to guide resuscitations and can avoid a significant proportion of futile thoracotomies [15].

Echocardiography is a very useful tool to evaluate the heart; abnormalities in wall motion, valvular disruption and pericardial effusions can be detected using this noninvasive technique. Actually, echocardiography is used as a complementary test in patients suspected of having a cardiac complication after a thoracic trauma [16].

The most common technique is the transthoracic echocardiography (TTE); this is an exam that requires a higher level of expertise and personnel and for these reasons is performed mainly by cardiologists. TTE is the first-choice technique to establish the diagnosis of cardiac tamponade [16].

Although TTE is widely accepted as one of the one of the first-choice tests for cardiac evaluation, sometimes its use may be limited by the patient’s anatomy or by traumatic abnormalities (such as fractured ribs, chest deformity, emphysema, abdominal distension, pneumothorax, pneumomediastinum, etc.). In this case, if echocardiographic data are requested, then the transesophageal approach can be used (TEE).

In TEE, a small ultrasound transducer connected to a flexible endoscopy is placed into the esophagus; this technique is very useful in the case of cardiac injury, such as pericardial effusion, cardiac tamponade, and myocardial contusion. It is also used for aortic injuries [16,17,18].

An anteroposterior plain chest radiography (CXR) is one of the primary diagnostic tools for detecting major thoracic injury or life-threatening intrathoracic pathology. The clinician can assess the pleural spaces, the mediastinum, and the bones, in addition to checking the position of the endotracheal tube. However, it is known that CXR has poor sensitivity when it comes to identifying some serious intrathoracic pathologies, including pulmonary contusions.

In trauma patients, contrast-enhanced computed tomography (CT) of the chest is frequently used as a more sensitive diagnostic tool. Currently, it represents the “gold standard” imaging technique in the first-line evaluation of polytraumatized patients.

In patients with severe blunt chest trauma confirmed by CT, 65% of significant intrathoracic injuries cannot be found by CXR. Although the CT can provide more accurate information on injuries, it should not be used indiscriminately; in fact, clinicians can use established rules and algorithms to determine when a chest CT scan is necessary.

Chest CT has become the routine procedure when numerous rib fractures are suspected or diagnosed by CXR. It is also used to identify all the chest wall or intrathoracic injuries, and to evaluate whether surgery is necessary.

CT is considered immediately for adults with suspected chest trauma without severe respiratory compromise who are responding to resuscitation or whose hemodynamic status is normal or stable [10].

## 2. Emergency Department Thoracotomy

Emergency Department Thoracotomy (EDT) is a surgical procedure for the acute treatment of traumatic cardiac arrest (TCA) recommended by the guidelines of the European Resuscitation Council (ERC), the Eastern Association for the Surgery of Trauma (EAST) and by many other trauma practice management guidelines [19].

The use of this technique is still controversial and there has been much debate about the indications for this procedure; the use has been documented in several clinical settings, such as penetrating and blunt thoracic injuries, cardiac injuries, thoracic traumas where the pulmonary hilo is involved, gaseous embolism or bronchopleural fistulas and vascular injuries in patients arriving in the Emergency Department (ED) with cardiopulmonary arrest [19].

Instead, anterior thoracic hemorrhages are treated by cross-clamping of the descending aorta [14].

The decision to perform EDT is strictly dependent on the mechanism of injury or wounding. Once stabilized, the patient should undergo surgery treatment.

The survival rate after EDT ranges from 35% for patients arriving in shock with a penetrating cardiac wound to 15% for all patients with penetrating thoracic injuries, with a decrease of 2% in patients with blunt trauma, and less than 1% survival in patients with no vital signs [20,21,22].

### 2.1. Indications

The type of trauma, location of the most severe injury and patient’s vital parameters are three fundamental indications for emergency thoracotomy.

In 2009, indications for EDT were reported [23]: (a) hypotension (systolic blood pressure < 70 mmHg) unresponsive to fluid resuscitation and/or inotropic support due to major intrathoracic hemorrhage (>1500 mL from chest drain), cardiac tamponade, gaseous embolism or bronchopleural fistulas; (b) cardiac arrest after isolated chest penetrating trauma with signs of life; (c) cross-clamping of the thoracic aorta to stop abdominal hemorrhage as a pre-laparotomy rescue procedure [19].

The contraindications for EDT were (a) the absence of signs of life; (b) cardiac arrest in a non-shockable rhythm for over 5 min. For patients with blunt thoracic trauma and abdominal vascular injuries, indications to EDT were revised during the last decades [19,24].

The best effectiveness is obtained in the case of penetrating wounds (8–10%), while the least effective is with blast wounds (about 1%); penetrating wounds have a higher survival rate (18–24%) than gunshot wounds (4–5%) [22,25]. The single site of penetrating lesion also correlates with a higher probability of survival compared to multiple sites [26,27]. In case of limb trauma or isolated abdominal wounds, the execution of ET should be carefully evaluated [28,29]. Of extreme importance is the evaluation of pulse, pupillary reflex, blood pressure and gag reflex. The presence of vital signs during roadside assistance or their loss within the first ten minutes after hospitalization correlate with a higher chance of survival in patients suffering from penetrating wounds. Patients with blunt or burst trauma lacking vital signs have a low survival rate and the EDT is not recommended in these cases.

As stated in the American guidelines, EDT is a valid aid in patients with electrical cardiac activity and penetrating thoracic trauma [30].

According to the systematic review conducted by Seamon et al. [31], EDT is strongly recommended in patients who present pulseless to the Emergency Department with signs of life after penetrating thoracic injury. In patients who present pulseless and without signs of life after a penetrating thoracic injury, the procedure has a conditional recommendation, as well as patients pulseless, with signs of life after a penetrating extra- thoracic injury or after a blunt injury.

In both the works [30,31], the use of EDT is not recommended in patients with a blunt injury and without signs of life.

Note how pericardiocentesis is not considered useful for these patients as it is unable to remove clots from the pericardium, is often accompanied by complications and delays the potentially life-saving surgical procedure.

During the management of a traumatic cardiac arrest, the airway should be secured with 100% oxygen provided [32].

### 2.2. EDT versus REBOA for Non-Compressive Torso Hemorrhage

In the last few years, resuscitative endovascular balloon occlusion of the aorta (REBOA) has gained popularity among trauma surgeons for the management of non-compressible torso hemorrhage.

REBOA is a procedure that involves the placement of an endovascular balloon in the aortic lumen to limit distal hemorrhage avoiding the morbidity of thoracotomy [33,34].

Although REBOA is not always accessible, it has recently been suggested as an alternative to EDT. Most of the data about this technique are from clinical series of patients who have experienced blunt or penetrating trauma; there is currently insufficient evidence to support its efficacy in reducing mortality rates. The benefits of EDT and REBOA in terms of mortality reductions for trauma patients are not well understood [35].

### 2.3. Procedure

Once EDT is indicated, the most rapid technique is a left anterolateral thoracotomy performed in the fourth intercostal space. This procedure was first described in 1904 by Spangaro [36]. Upon opening the pleural cavity, a thoracic retractor will be placed which will allow, once hemostasis is obtained, the pleural cavity to be exposed looking for the main lesion. If necessary, the thoracotomy can be enlarged by means of a transverse sternotomy.

In the event of injury to a large vessel, an attempt should be made to stop the bleeding by pressing and subsequent closure and ligation. In the event of a gas embolism, the pulmonary hilum must be blocked, or the affected lung must be rotated by 180 in order to evacuate the air from the aorta. If hemopericardium is present, the pericardial sac can be opened in the cranio-caudal sense, from the aortic root towards cardiac apex. Once the clots have been removed, it will be possible to identify the source of bleeding and carry out a manual compression; it will thus be possible to attempt to carry out a first hemostasis with sutures.

In patients with suspected hemorrhage in the right chest, a clamshell incision can be required [37]. The clamshell incision or bilateral transverse bithoracosternotomy was first described in 1991 by Pasque and Cooper [37]. This technique represents an alternative to median sternotomy and guarantees excellent access to the pleural spaces.

Another method that can be used in an emergency is the introduction of a Foley catheter into the hole which will be inflated to stop bleeding [38]. By moving the lung forward and exposing the posterior mediastinum, it will be possible to access the descending aorta for its possible clamping or manual compression. This allows for the improvement of the afterload, increased coronary and cerebral perfusion, and reduced infra-diaphragmatic bleeding [32].

The hemodynamic effect of aortic cross-clamping during EDT can be achieved by a transfemoral insertion of a balloon catheter called REBOA [33,34].

In some cases, to facilitate the identification of the flaccid-descending thoracic aorta due to hypovolemia, a nasogastric tube can be introduced, and mechanical ventilation suspended. The mediastinal pleura is accessed by the posterior approach and a cross-clamp is placed, thus restoring volemia and allowing the execution of the intervention.

### 2.4. Management of Specific Injuries

#### 2.4.1. Blunt Thoracic Aortic Injury (BTAI)

The most common anamotic site of injury in BTAI occurs at the thoracic isthmus, on the median luminal aspect of the descending thoracic aorta, distal to the origin of the left subclavian artery [39].

Aortic injuries are graded in terms of the layers involved in the injury and the grades are important because they lead the management, ranging from the conservative Grade 1 to surgery/endovascular approach to Grade 2 and 3.

Thoracic endovascular aortic repair (TEVAR) is the first-choice treatment method, especially in patients with aortic injury resulting from blunt trauma; however, there have also been successful cases of penetrating TAI treated in this way.

Numerous studies have described TEVAR’s benefits over open repair; however, no randomized control trials have been conducted [40,41].

The advantages of TEVAR include lower mortality, no need for cardiac bypass, less risk of ischemia, blood loss and shorter operating and hospital length of stay.

On the other hand, TEVAR has disadvantages, such as the requirement for specialized personnel to perform the procedure, the size of the vessel needed for the access and the graft’s lumen size, which depends on the hemodynamic status [39,41].

#### 2.4.2. Penetrating Thoracic Aortic Injury (PTAI)

PTAIs are less common then BTAIs and are most frequently due to gunshots and stabs.

PTAIs are usually lethal and patients have very severe conditions due to rapid exsanguination. If the patients are hemodynamically unstable, the only option is to take them to the operating room for open surgery; if the patients are stable, because of the foreign body in blood vessel tamponade, then the endovascular approach is possible via stenting or endograft [39].

#### 2.4.3. Diaphragmatic Injuries and Video-Assisted Thoracoscopic Surgery (VATS)

Anytime there are wounds below the level of the nipples, it is important to suspect injuries that involve the diaphragm and the abdomen.

VATS can be used to assess thoracic injuries on the left side; a reparation/stabilization of the chest wall is also possible with VATS. Right-sided diaphragmatic injuries are typically shielded from herniation and damage to the gastrointestinal tract by the liver.

If there is no suspicion of surgical thoracic injuries, the laparoscopic approach is recommended; this avoids the need for double lumen intubation [9].

### 2.5. Intrathoracic Injuries

The most common injuries are penetrating wounds to the pulmonary hilum and great vessels. Intrathoracic hemorrhage control may require hilar cross-clamping.

If there is an important hilar hematoma with active bleeding, twisting the lung will occlude both the pulmonary artery and pulmonary veins. This technique is very complicated, because the risk of damaging the bronchus during the maneuver is high, and it can produce several damages to the lungs and is not well tolerated in hypotensive patients.

If the pulmonary parenchymal hemorrhage is away from the hilum, a non-crushing vascular clamp is preferred to stop the bleeding.

Treatment for a broncho venous air embolism requires immediate pulmonary hilar cross-clamping to prevent the propagation of pulmonary venous air. The Trendelemburg position can be helpful to trap hair to the apex of the left ventricle, and then can be removed by needle aspiration [19].

### 2.6. Esophageal Injuries 

Esophageal injuries are classified according to the mechanism on injury in penetrating (firearms or stab wounds) and blunt trauma, and according to its anatomic location in cervical, thoracic and abdominal esophageal injuries.

The first step for its management is the securement of the airways; the patients must remain nil per os (NPO) with a nasogastric tube placed by endoscopy to prevent the extraversion of the gastric material. It is mandatory to start immediately a full spectrum antibiotic coverage.

After the evaluation of the type of injury and the site of the lesion, sometimes the management can be non-operative; in this case, the patients require intense monitoring, NPO status, placement of an NG tube and enteral feeding via. a feeding tube, or nutritional support with TPN and drainage to potential collections.

On the other hand, there are many types of surgical approaches for this type of injuries, which change based on the site of the injury and on the patient’s condition. The surgical management is well described in the review written by Petrone et al. [42] (Table 1).

## 3. Urgent Thoracotomy

Urgent thoracotomy is a procedure performed in the first hours after injury. It is used for the treatment of non-bleeding lesions of the great aortic vessels (less often due to traumatic rupture at the thoracic level), cardiac lesions, tracheobronchial lesions and esophageal lesions. The presence of cardiac tamponade, persistent air loss, gas embolism and abundant bleeding from the drainage are all indications for urgent thoracotomy. It is used to treat injuries to the aorta, esophageal and tracheobronchial lesions.

Often the indication for surgical exploration is based on the observation of the thoracic drainage. The drainage must be placed every time the physical examination or radiography suggests a hemothorax. The next question to ask is if and when the bleeding will stop. If large amounts of blood are drained, then a vascular lesion that is difficult to resolve without surgery should be suspected. In the literature, drainage of 1500 mL of chest drainage represents the limit quantity for mandatory surgical exploration. In common clinical practice, the limit is set at 1000 mL [68,69,70]. It is also an indication for thoracotomy if the bleeding from the drainage tube is maintained at a rate of 200 to 300 mL/h [6,69,70]. These guidelines do not always apply to all patients.

Chest drainage should be viewed critically for (1) parenchymal lung hemorrhage, (2) pneumothorax, (3) blunt thoracic trauma with nuanced symptoms and (4) coagulopathic patients, especially in elder age. The bleeding of the pulmonary parenchyma is conditioned by the very nature of the pulmonary circulation, characterized by low pressure that allows, through full lung expansion, to prevent parenchymal hemorrhage. Likewise, patients can also present with delayed closed thoracic trauma. This patient may present with significant hemothorax but has slowly built up over the time it took to reach the emergency room. In these situations, if we evaluated the amount of blood drained and did not consider the time factor, we could put a wrong indication for a surgical exploration. This obviously does not apply to patients suffering from penetrating trauma or bruises. In these cases, the time between exposure to the trauma and treatment is very short; for this category of patients, 1000–1500 mL of chest drainage is a solid parameter for chest exploration. Thoracotomy must be carefully evaluated in thoracic trauma when it is associated with a coagulopathy. Two typical examples are the elderly population taking anticoagulant drug therapy and patients with coagulopathy suffering from blunt head injury. According to the authors, in these situations, the surgery is not only not therapeutic but can increase the already-present bleeding of the chest. Another important indication for urgent exploration of the chest after trauma is the presence of a large amount of air loss defined as present and persistent in all phases of mechanical respiration associated with the inability of the lungs to expand and ventilate due to the loss of effective tidal volume. This usually occurs in the presence of major tracheobronchial lesions. Pericardial window or median sternotomy are techniques used in case of cardiac tamponade, which is semiotically characterized by Beck’s triad (muffled heart tones, dilated neck veins and hypotension), with pericardial effusion at ultrasonography. Air embolism is the result of a communication between a bronchus and a pulmonary vein, and it is infrequent after penetrating wounds; in this case, an urgent thoracotomy is required. Following the application of positive pressure ventilation, the patient, if hemodynamically stable, may present neurological deficits or acute heart failure [71].

In conclusion, the main indications for emergency thoracotomy are (1) drainage of more than 1000 mL, (2) evidence of persistent blood loss of 200–300 mL/h, (3) massive air loss, (4) tamponade cardiac and (5) air embolism.

### Surgical Approaches

Once the right indication to intervention has been placed, the choice of the incision of the chest is of fundamental importance. It is necessary to consider the coexistence of four different anatomical compartments: the right hemithorax, the left hemithorax, the mediastinum and, if there is a lesion of the abdomen, the peritoneal cavity. It is necessary to consider the trajectory of the penetrating object (for example, direction of the bullet) and in consideration of the anatomical areas crossed, choose the most suitable surgical route. Table 2 lists the most common surgical approaches for thoracic viscera injuries. For example, a bullet that pierces the chest anteriorly in the left parasternal region can damage the entire left ventricle wall. A surgical treatment approach is consistent with the site of the injury; thus, for instance, anterior lesions are easily amended by anterior thoracotomy and posterior ones by left postero-lateral thoracotomy.

Parasternal stab wounds are usually superficial, mostly involving the anterior mediastinum. For these wounds, median sternotomy is usually the most appropriate. Sternotomy offers an optimal visualization of the heart cavities and major vessels, especially in the case of gunshot wounds at the right parasternal level; for a better exposure, the sternotomy can be extended cranially. In left parasternal gunshot wounds, the approach consists in a posterolateral thoracotomy, to better expose the posterior portion of the heart. Lateral wounds, mostly posterior, indifferently to the right or left, should be treated with posterolateral thoracotomy. Posterolateral thoracotomy offers an excellent view of the great vessels, such as the subclavian artery or aorta in the case of massive hemothorax. In cases of air embolism and massive air loss, left posterolateral thoracotomy is the gold standard approach. Lesions of the right lung of the trachea and proximal lesions of the left main bronchus are excellently treated with right thoracotomy. Right thoracotomy is the main access route for most esophageal lesions, except distal ones which are best accessed through the left side. Right thoracotomy exposes the cardiac atria and the right ventricle. Left thoracotomy is used to expose left pulmonary structures, aorta, proximal left subclavian artery, left heart chambers, distal esophagus, and left distal main bronchus. A “clamshell” incision or bilateral anterolateral thoracotomy in the fourth or fifth intercostal space provides excellent access to right heart cavities, while offering a poorer exposure of any other thoracic structure.

## 4. Urgent Exploratory Thoracotomy

Urgent exploratory thoracotomy, performed at the fifth intercostal space homolateral to the lesion, is recommended in cases of diagnostic doubt. If possible, consider the use of a double lumen endotracheal tube for lung collapse. In the presence of an ascertained diagnosis, an incision will be performed instead, capable of providing optimal exposure. The surgeon’s experience plays an important role in choosing the access route. The inexperienced surgeon is led to repair the lesion with a non-optimal exposure; once the risk of death has been averted, it is often necessary to enlarge the thoracotomy or perform a second surgical access for the definitive repair of the lesion. Finally, a thoracotomy for packing venous bleeding of the lung or chest wall is a common approach that is too often overlooked. The inspection of the pleural cavity after trauma, performed by means of an exploratory thoracotomy, should be conducted through a systematic sequence of surgical, logical, and orderly gestures. First, all clots must be removed from the pleural cavity and then the lung must be mobilized (also through the section of the lower ligament) in order to free the surgical field; in this way, it is possible to explore the mediastinum and the pericardium in search of bleeding. A hemopericardium may not be visible to the naked eye so it is recommended to make a small incision of the pericardium which can be enlarged if necessary. Direct control of the pulmonary hilum allows us to dominate any bleeding of the great vessels both proximally and distally. Pulmonary parenchymal bleeding can also be dominated by having direct access to the pulmonary hilum. After the bleeding has been checked, the damaged structures can be repaired definitively.

## 5. Delayed Thoracotomy

A deferred thoracotomy (>24 h) can be performed in the absence of tracheobronchial lesions, traumatic rupture of the aorta, intracardiac lesions, retained hemothorax and post-traumatic empyema. Typical manifestations of underdiagnosed tracheobronchial lesions are tracheal/bronchial stenosis or persistent air leakage. Traditionally, immediate repair is always favored for traumatic rupture of the thoracic aorta. However, since a thorough knowledge of the natural history of these lesions and of pharmacological treatment has been acquired over time and with experience, the concept of deferred repair is becoming more and more widespread [72,73,74,75]. The immediate treating of the most life-threatening injuries is the primary goal of the delayed repair, by avoiding systemic heparinization in the hours immediately following the trauma [76]. The early use of beta-blockade allows selective aortic repair from 2 to 29 months after severe abdominal trauma, significant pulmonary contusions or cerebral hemorrhages [77]. Similarly, immediate repair is necessary when patients develop severe congestive heart failure, whereas intracardiac injuries, such as ventricular septal defects or atrioventricular valve insufficiency can be approached by delayed repair [5].

Of fundamental importance is the thoracic drainage procedure for hemothorax. This maneuver must be carried out in absolute sterility even if in emergency conditions. The persistence of the hemothorax or superinfection with the appearance of empyema is the consequence of improper positioning of the drainage. An incompletely drained hemothorax can undergo different outcomes: healing, fibrothorax or post-traumatic infection [78]. Morbidity is not reduced by use of antibiotics, even when administered in prophylaxis [79]. Empyema is most frequently caused by blood retention in the pleural space; so, it is essential to do everything possible to drain the pleural cavity in the best possible way and sterile, taking care to quickly remove the tubes first. If hemothorax cannot be evacuated by a well-placed chest tube, thoracotomy or video-assisted thoracoscopy, when possible, may be considered. If there are clots in the cavity, chest drainage is often not effective [80,81,82]. Often the evaluation of a hemothorax with a chest X-ray can be misleading as this examination tends to underestimate the amount of spilling in the chest cavity. CTs can accurately measure the extension of the hemothorax and are useful for choosing the most appropriate surgical approach [83]. A relatively easy technique, both to perform and in terms of efficacy, is minimally invasive video-assisted thoracoscopy [84,85,86]. Approximately 20% is converted to thoracotomy, and blunt trauma is the most frequent cause, the second being a video-assisted thoracoscopy performed a few days after the trauma [80,81,82]. When hemothorax is chronic, or maintained by continuous oozing by untreated rib fractures, thoracotomy is almost always necessary. In these cases, the intervention is longer and more complicated due to the presence of adhesions [87]. There is often bacterial superinfection [78]. A posterolateral thoracotomy is the most appropriate surgical approach in these cases.

Moreover, if the hemothorax is coagulated, it has a fibrous layer that needs to be removed. The fundamental point of this intervention is the complete removal of the pleura visceral layer to obtain a full re-expansion. After that, the pleural cavity should be adequately drained to prevent blood from re-accumulating and to ease the re-expansion of the affected lung. Finally, chest tubes should stay inserted only for the necessary time. Thoracic trauma, due to the particular anatomical site and the presence of important organs in its context, can lead to very serious short- and long-term sequelae, such as severe disability and death. Considering the worldwide case history of all traumas, thoracic traumas are the third most common cause of death in polytraumatized patients, acting as a contributing factor to death in 25% of cases [88]. Death can occur by several mechanisms, such as airway obstruction, respiratory tree lesions or massive bleeding. For example, penetrating chest trauma, usually the result of stab wounds, is counted among the most potentially lethal. Penetrating lesions of the heart present a high mortality, especially due to the hemodynamic instability that is configured, while injuries to large vessels such as the aorta can result in pictures that can quickly lead to shock. In pericardial lesions, in case of small laceration, there is less risk of mediastinal lesions and above all the blood is less likely to escape into the pleural cavity; cardiac tamponade occurs when the amount of bleeding is equal to a volume of 150–200 mL. When the pointed medium is very sharp and has a small section, the continuous solution is minimal or even point-like, and very difficult to identify. However, these are lesions that can have an equally lethal effect if they affect large blood vessels or the heart wall with consequent serious bleeding and cardiac tamponade. Such cases demonstrate the importance of carrying out a thorough external examination of the corpse, not neglecting minimal, but potentially fatal injuries. In cases of penetrating injuries with probable judicial drift, it will be important for doctors to document the site of injury and its extension, which can provide valuable help to the judicial authority and to any medico-legal investigations.

Similarly, blunt trauma is the most common cause of mortality, as it often results from road traffic accidents, mostly involving the young population. This figure clearly reflects global technological and demographic progress, not followed by adequate planning in terms of public safety (the significant increase in the number of vehicles circulating, the poor control of compliance with the safety measures imposed by law (speed limits, safety distance, restraint systems, road signs), distracted or unsafe driving due to the intake of alcohol in excessive quantities or hallucinogenic substances or narcotics). The high morbidity and mortality of this type of trauma lies in the fact that it can lead to simultaneous damage to several organs, with related complications that make the prognosis poor [89,90]. With regard to the finding of thoracic injuries or in any case capable of justifying the death or a serious illness of the driver, the considerable difficulty of reaching absolutely clarifying medico-legal assessments is emphasized, also due to the fact that, although the trauma can intervene immediately after the illness or immediately after the death of the driver, in any case, it determines lesions with characters practically indistinguishable from those produced in life. It must also be considered that the presence of extensive and widespread traumatism can sometimes mask a pathological pre-existence [91,92]. Literature [93] showed that chest trauma from intense deceleration can produce a rupture of the aorta with possible association of the mechanism of “whiplash” on the dorsal spine, especially at the level of the segment in which the aortic arch joins with the descending tract. The aortic rupture would be due to the pendulum effect of the heart inside the rib cage, made possible by the elasticity of the endothoracic viscera. By inertia, the cardiac mass continues in its forward movement stretching the vascular structures that depart from its base, the most rigid of which is precisely the aorta and above all the distal end of the aortic arch, which is adhered to the dorsal spine. The aortic rupture is often clear and circular; sometimes, complex lesions are observed characterized by transverse lacerations of the intima that depart from the principal rupture with arrangement like the rungs of a ladder (tearing in the shape of a ladder or ladder tears). Such lacerations can be found independently of a real rupture of the aortic wall, with dissection of the wall and death after a few hours or days from the accident. Cardiac lesions can occur even in the absence of clear traumatic signs of the sternum or rib cage, especially in relatively young subjects, in whom the osteocartilaginous structures.

## 6. Medico-Legal Implications of Thoracic Trauma

About 50% of polytraumatized patients reports a serious chest injury that represents the main cause of death. For the importance of the organs located in this area, death occurs both because of the trauma itself (acute), and after some time due to the onset of infections, fibrothorax, etc. (secondary damage).

Blunt injuries can cause rib fractures. Commonly, rib fractures in the adult cause no symptoms other than pain. If multiple rib fractures are present, the functional integrity of the chest wall may be compromised and it is manifested by a paradoxical breathing or, if the sharp ends of the fractured ribs are pushed inward, they can cause a pneumothorax. If blood vessels are injured, the trauma can be associated with hemothorax.

Rib (and sternal) fractures in adults, often identified during post-mortem examination, can be the sign of cardiopulmonary resuscitation (CPR). They are often symmetrical and, if no restoration of perfusion is achieved after the CPR attempts, they are not associated with hemothorax.

The nature of rib fractures, that are thought to be related to CPR, can become the subject of discussion in the court and, in an individual with a chest trauma history and who has received CPR for a prolonged period, it may be helpful to perform post-mortem microscopic examination to identify the nature of these fractures (either of traumatic or resuscitation origin).

Penetrating chest injuries, when caused by an acute trauma (stab wounds, gunshot wounds) can damage any of the organs or the chest cavity. The effect will depend on the damaged anatomical compartment. If the vessels are damaged, a thoracic hemorrhage will occur. When the hemorrhage is internal and there are no external signs of bleeding, it is difficult to identify the trauma in vivo, while liters of blood are frequently found in the thoracic cavity during the autopsy. In these cases, it is difficult to understand the origin of the bleeding and to interpret the crime scene; for example, when no traces of blood can be found during site inspection, this could mean that the wound was inflicted somewhere else [94].

Because of the interpretative difficulties that you can encounter in the evaluation of useful elements, also considering the absence of defined criteria, it is necessary to emphasize the need for a scrupulous and rigorous description of the objective data and the meticulous collection of every single judgment element, starting from the site inspection which, therefore, is a key point in forensic practice [95].

Then, the investigation continues in the autoptic room where, starting from the collection of clothes, continuing with the interpretation of the external and internal lesions on the body, the forensic pathologist proceeds to identify the nature and characteristics of the collected findings and the means that produced them, reconstructing the dynamic of the event (homicide, suicide, work accident, self-harm, accident) to assess whether the event that has occurred (trauma) is compatible with the lesions.

From a legal point of view, traumatology is a subject of controversy, also in the field of medico-legal litigation.

In fact, when the on-call doctor is faced with a criminal event, he is required to inform the judicial authority by issuing a “report” or “reference”. It is up to the healthcare professional to ascertain if the case has the characteristics of a crime punishable ex officio (homicide, personal injuries, sexual crimes, tampering with a corpse, intoxications, abuses).

For this reason, it is sometimes necessary to carry out toxicological tests which may be relevant for the purposes of the criminal proceeding. The law has established that blood sampling, carried out for medical purposes during hospitalization in a public hospital, is usable against the defendant in criminal matters because they are documentary evidence. On the other hand, if these investigations are carried out for an explicit request of the Judicial Police, the defendant can refuse the blood sample and so, the possible assessment is unusable for the purposes of the assertion of responsibility, even if by means of sampling by the healthcare staff.

In the case of blood sampling for first aid/live-saving therapies, such as mediastinal surgery or the surgery of the great thoracic vessels cause of hemothorax, signed informed consent is not relevant; on the contrary, if the withdrawal is necessary for the verification of the alcohol level or the use of drugs, the possibility to be assisted by a lawyer must be notified to the suspect. In the absence of this advice, the examinations that are carried out are unusable in the courtroom.

In the early 19th century, toxicology developed in the forensic field too. Its purpose is to identify, through specific analytic methods (immunohistochemistry, spectroscopy, gas chromography) a cause–effect relationship between the presence of a toxicological substance and a person’s death. Today, forensic toxicology is also essential to identify the guilt of a defendant in the case of homicide, suspected suicides, driving under the influence of alcohol, etc.

In conclusion, traumatology is a very delicate subject both in the legal and specifically forensic fields. Thoracic traumas, depending on the delicate body area they affect, represent the most critical branch of traumatology both for what concerns the offered treatment and the ways in which this treatment is carried out, and for the medico-legal consequences that they involve.

## 7. Conclusions

The nature of lesions and their criticality affect the choice of timing and type of surgery. The site of the injury, together with the type of trauma, dictate the choice of surgical technique and the relative access.

There are many variables to consider while in the act, without neglecting the peculiarities of each trauma. Some of the most common traumas and the various scenarios that may be encountered are here described to suggest general guidelines for actual management. Different surgical approaches have been gathered by our experience. Patients with chest trauma are at a high risk of death and direct complications. A surgical intervention performed by learned and experienced professionals is essential to obtain favorable results for patients.

The emergency management of thoracic traumatology also requires a careful study of the patient’s clinical conditions and above all, in cases that require an immediate approach, the timeliness of action will also be fundamental in order to avoid litigation, also given the importance from the pathological and epidemiological point of view of the structures possibly involved.

## Figures and Tables

**Table 1:** Principal injuries.

Type of Trauma	Sign and Symptomps	Diagnosis	Treatment
Cardiac injuries [5,43,44]	Pericardial tamponade, haemorrhagic shock. (mortality: 10–70%)	Pericardiocentesis.	Sternotomy
Great vessels injuries [45,46,47,48,49]	Hemothorax, pericardial tamponade, pulseless, stroke, coma.	Thoracotomy, angiography, CT-angiography.	median sternotomy (subclavian or parasternal lesions; ascending aorta); posterolateral thoracotomy (subclavian artery; vertebral and mammary arteries); patch venography
Pulmonary injuries [7,8,49,50,51,52,53,54,55,56,57,58,59]	Pneumothorax, hemothorax, shock. (mortality: 40–50%)	CT.	Chest tube; thoracotomy for anatomical lung resections; pneumonectomy
Tracheobronchial injuries [60,61]	Subcutaneous emphysema, hemoptysis, stridor, respiratory distress, hypertensive pneumothorax, pneumomediastinum	Bronchoscopy, CT.	Thoracotomy; drain placement
Esophageal injuries [42,44,62,63]	Hematemesis, odynophagia, dysphagia.	Chest X-ray, CT, surgical exploration, endoscopic examination.	Nasogastric tube position by endoscopy+ antibiotic cover; enteral feeding via feeding tube or nutritional support with TPN; cervicotomy; right posterolateral thoracotomy (upper and middle injuries); left posterolateral thoracotomy (distal injuries), esophageal deviation, esophageal exclusion, esophagectomy, T tube drainage, Endo-VAC therapy
Traumatic rupture of the thoracic aorta [64,65,66,67]	There is no specific sign or symptom for such injuries. (mortality: 90%)	Chest X-ray, CT angiography	Beta blockers, endovascular stent, primary anastomosis requires cardiopulmonary bypass

**Table 2:** Surgical approaches from traumatic injuries to thoracic viscera.

Site	Sternotomy	Right Thoracotomy	Left Thoracotomy
Right atrium	+++	++	0
Right ventricle	+++	+	+
Left atrium	+++	+	+
Left ventricle	++	0	+++
SVC	+++	++	0
Azygos vein	++	+++	0
IVC	+++	++	0
Aortic root	+++	+	0
Aortic arch	+++	0	++
Right subclavian	++	++	0
Proximal right carotid	+++	+	0
Innominate	+++	++	0
Left subclavian	+	0	+++
Proximal left carotid	++	0	++
Descending aorta	0	+	+++
Main PA	+++	0	++
Right PA	++	+++	0
Left PA	++	0	+++
Right UL	++	+++	0
Roght ML	++	+++	0
Right LL	+	+++	0
Left UL	+	0	+++
Left LL	0	0	+++
Right hilum	++	+++	0
Left hilum	++	0	+++
Pericardium	+++	++	++
Right IMA	++	+++	0
Left IMA	++	0	+++
Proximal esophagus	0	+++	0
Distal esophagus	0	++	+++
Proximal trachea	++	+	+
Carina	0	+++	+
Right main stern	0	+++	0
Lest main stern	0	++	++
Right hemidiaphragm	+	+++	0
Left hemidiaphragm	+	0	+++
CPB	+++	++	++

Abbreviations: CPB, cardiopulmonary bypass; IMA, internal mammary artery; IVC, inferior vena cava; LL, lower lobe; ML, middle lobe; PA, pulmonary artery; SVC, superior vena cava; UL, upper lobe. +++, preferred; ++ acceptable; +, with difficulty; 0, not accessible; Data from Ritchie WP, Steele G, Dean HR, editors. General Surgery. Philadelphia: JB Lippincott; 1995 p.861 [5].

## Data Availability

Not applicable.
